# Supplementary material for: Association of the Dietary Inflammatory Index with Depressive Symptoms among Pre- and Post-Menopausal Women: Findings from the National Health and Nutrition Examination Survey (NHANES) 2005–2010
Source: Nutrients. 2022 May 9;14(9):1980. doi: 10.3390/nu14091980 (PMC9105364; doi:10.3390/nu14091980)
Supplement: Supplementary file 1 [file nutrients-14-01980-s001.zip › nutrients-1651031-supplementary.pdf]

Table S1. Estimated Beta and Standard Errors for the Effects of Dietary Inflammatory Index Quartiles and Score for Somatic Symptoms of Depression* among Pre- and Post-menopausal Participants from the National Health and Nutrition Examination Survey 2005–2010.

	Model 1 [a]		Model 2 [b]	
	**Beta (SE)**	**95% CI**	**Beta (SE)**	**95% CI**
	Pre-menopause (*n* = 2512)			
Quartile 1	Referent	–	Referent	–
Quartile 2	0.33 (0.15)	0.02, 0.64	0.19 (0.15)	−0.10, 0.49
Quartile 3	0.46 (0.15)	0.16, 0.76	0.20 (0.15)	−0.09, 0.49
Quartile 4	0.76 (0.14)	0.47, 1.05	0.31 (0.15)	0.02, 0.61
p-trend	<0.001		0.050	
	Post-menopause (*n* = 2392)			
Quartile 1	Referent	–	Referent	–
Quartile 2	0.26 (0.15)	−0.4, 0.56	0.10 (0.15)	−0.19, 0.40
Quartile 3	0.39 (0.15)	0.09, 0.68	0.09 (0.15)	−0.20, 0.38
Quartile 4	0.83 (0.14)	0.54, 1.11	0.37 (0.15)	0.08, 0.67
p-trend	<0.001		0.010	

* Range of Score: 0–12 [a]. Model 1: Adjusted for age [b]. Model 2: Adjusted for age, race (White, Hispanic, Black, Multiracial), BMI (underweight/normal weight, overweight/obese), waist circumference (≤35 inches (low risk), >35 inches (high risk)), marital status (Married/Partner, Divorced/Widowed/Separated, Never married), education (below high school, high school degree, some college/AA, college graduate and higher), smoking (never smoker, ever smoker), poverty-to-income ratio (quartiles), physical activity (quartiles). SE, standard error; CI, confidence interval; BMI, body mass index; AA, associate degrees.

Table S2. Estimated Beta and Standard Errors for the Effects of Dietary Inflammatory Index Quartiles and Score for Cognitive Symptoms of Depression* among Pre- and Post-menopausal Participants from the National Health and Nutrition Examination Survey 2005–2010.

	Model 1 [a]		Model 2 [b]	
	**Beta (SE)**	**95% CI**	**Beta (SE)**	**95% CI**
	Pre-menopause (*n* = 2512)			
Quartile 1	Referent	–	Referent	–
Quartile 2	0.31 (0.15)	0.1, 0.61	0.18 (0.15)	−0.10, 0.48
Quartile 3	0.53 (0.14)	0.25, 0.82	0.29 (0.14)	0.007, 0.58
Quartile 4	0.81 (0.14)	0.53, 1.09	0.42 (0.14)	0.13, 0.71
p-trend	<0.001		0.003	
	Post-menopause (*n* = 2392)			
Quartile 1	Referent	–	Referent	–
Quartile 2	0.21 (0.15)	−0.8, 0.51	0.05 (0.15)	−0.24, 0.34
Quartile 3	0.37 (0.14)	0.09, 0.66	0.04 (0.14)	−0.24, 0.33
Quartile 4	0.87 (0.14)	0.59, 1.15	0.32 (0.14)	0.04, 0.61
p-trend	<0.001		0.016	

* Range of Score: 0–15 [a]. Model 1: Adjusted for age [b]. Model 2: Adjusted for age, race (White, Hispanic, Black, Multiracial), BMI (underweight/normal weight, overweight/obese), waist circumference (≤35 inches (low risk), >35 inches (high risk)), marital status (Married/Partner, Divorced/Widowed/Separated, Never married), education (below high school, high school degree, some college/AA, college graduate and higher), smoking (never smoker, ever smoker), poverty-to-income ratio (quartiles), physical activity (quartiles).
